# Availability of Healthy Food and Beverages in Hospital Outlets and Interventions in the UK and USA to Improve the Hospital Food Environment: A Systematic Narrative Literature Review

**DOI:** 10.3390/nu14081566

**Published:** 2022-04-09

**Authors:** Sarah Richardson, Lorraine McSweeney, Suzanne Spence

**Affiliations:** Human Nutrition Research Centre, Population Health Sciences Institute, Faculty of Medical Sciences, M1.151 William Leech Building, Newcastle University, Framlington Place, Newcastle NE2 4HH, UK; sarah.richardson2442@outlook.com (S.R.); suzanne.spence@ncl.ac.uk (S.S.)

**Keywords:** food environment, healthy diet, hospital, systematic review, narrative synthesis

## Abstract

The aims of this systematic review are to determine the availability of healthy food and beverages in hospitals and identify interventions that positively influence the hospital food environment, thereby improving the dietary intake of employees and visitors. Embase, Medline, APA PsycInfo, Scopus, Google Scholar and Google were used to identify publications. Publications relating to the wider hospital food environment in the UK and USA were considered eligible, while those regarding food available to in-patients were excluded. Eligible publications (*n* = 40) were explored using a narrative synthesis. Risk of bias and research quality were assessed using the Quality Criteria Checklist for Primary Research. Although limited by the heterogeneity of study designs, this review concludes that the overall quality of hospital food environments varies. Educational, labelling, financial and choice architecture interventions were shown to improve the hospital food environment and/or dietary intake of consumers. Implementing pre-existing initiatives improved food environments, but multi-component interventions had some undesirable effects, such as reduced fruit and vegetable intake.

## 1. Introduction

Overweight and obesity are extremely prevalent across the UK and USA. In 2018, it was estimated that 67% of men and 60% of women in the UK had overweight or obesity [1], along with 71.6% of American adults in 2015/2016 [2]. A high body mass index (BMI) is linked to a range of non-communicable diseases, such as hypertension, type 2 diabetes and coronary heart disease [3], which has led to a significant number of hospital admissions associated with weight-related disorders. Between 2014 and 2015, it was estimated that the economic cost of overweight and obesity-related health complications to the National Health Service (NHS) was GBP 6.1 billion [4], while the healthcare cost of obesity in America was approximately USD 149.4 billion [5].

In addition to the high prevalence of obesity and overweight in the general population, healthcare employees demonstrate similar weight-management issues. One study carried out by Kyle et al. (2017) used data from the 2008–2012 Health Survey for England and found that 25.1% of the nurses surveyed had a BMI of 30 kg/m^2^ or higher, classifying them as ‘obese’. Furthermore, 32% of unregistered care workers, 26% of non-health-related NHS employees and 12% of other healthcare professionals also had a BMI of 30 kg/m^2^ or higher. These values are similar among American hospital staff members, with 27% of American nurses estimated to be obese [6].

A key cause of obesity is eating an excess of unhealthy foods. In the UK, the Office for Health Improvement and Disparities advises infrequent consumption of foods high in fat, salt and sugar [7]. American guidelines reflect the same general recommendations; according to the 2015–2020 Dietary Guidelines for Americans, a healthy diet should involve the restriction of saturated and trans fats, added sugars and salt [8]. Therefore, unhealthy foods can be defined as products that are high in these substances.

The range of healthy or unhealthy food and beverages available, food marketing techniques and the cost of food items in a specified setting can be referred to as the food environment [9]; this has a significant impact on the nutritional quality of food consumed by the general public. Studies have shown that there is an association between easier access to fast food and greater BMI and odds of obesity [10], suggesting that the food environment has a strong influence on weight status.

The general public has an expectation of hospitals and other healthcare environments to promote healthy behaviours, with 97% of participants in one survey indicating that hospitals should act as positive role models for healthy lifestyle behaviours [11]. Despite this, unhealthy foods are often found in hospital food outlets.

The aim of this review is to explore the extent to which healthy food and drink options are available to employees and visitors in hospital food environments and to determine which interventions are effective in reducing the purchase and consumption of unhealthy foods and beverages. This research may identify interventions that can improve the health and wellbeing of hospital employees and visitors, potentially leading to policy change to ensure healthy food is predominant in the wider hospital food environment.

## 2. Materials and Methods

The protocol associated with this systematic review was registered with PROSPERO, an international database of prospectively registered systematic reviews (reference number CRD42021223249). An amendment was made on 12 August 2021, detailing the repetition of database searching and a revised quality assessment method.

To find literature relevant to the current hospital food environment and interventions to improve the nutritional quality of food available to employees and visitors, a systematic search was carried out using five electronic databases: Embase, Medline, APA PsycInfo (all accessed via Ovid), Scopus and Google Scholar. Google was also utilised to ascertain suitable grey literature. Initial searches were carried out on 23 October 2020 and repeated on 21 July 2021 to detect new publications.

Suitable keyword search terms were identified; controlled search terms included “hospital”, “convenience food”, “healthy diet”, “automatic food dispensers” and “nutritional value”. Key words were amended slightly for each database; full search terms are listed in Appendix A.

Eligibility criteria were established to aid the selection of relevant publications. Some criteria were used to narrow the scope of the research to facilitate a detailed review of source material within the timeframe available. Two researchers carried out independent eligibility screening using Rayyan [12], and disputes were resolved via discussion with a third researcher.

Publications were included in the present review if they related to the wider hospital food environment (i.e., food outlets accessible to hospital employees and/or visitors) in the UK or the USA. No restrictions were placed on publication dates. Publications were excluded if they focused on food available only to patients in hospital wards. Additional exclusion criteria included studies with no full-text sources available, studies written in a language other than English and studies which involved systematic reviews or meta-analyses.

Search results were imported to Endnote [13]; duplicates were removed before the sample was exported to Rayyan [12]. Studies were initially screened based on the adherence of their titles and abstracts to the eligibility criteria. Included studies were further refined by screening full texts and removing ineligible records.

Several key pieces of data were extracted from each study in a standardised template by one researcher. Extracted information included author names, year of publication, country, study design, aim, duration, intervention/observation methods, outcome measures and results. A quality assessment and risk of bias analysis was also carried out on each source by one researcher using the Quality Criteria Checklist for Primary Research from the Academy of Nutrition and Dietetics [14]. See Appendix B, Table A1 for the full data extraction table.

The key outcomes of interest were the nutritional quality of food and beverages currently available to employees and visitors in hospitals as well as interventions aiming to improve the nutritional quality of products, awareness of nutritional values, dietary intake or overall health of hospital employees and visitors. Summary measures for these outcomes varied greatly between eligible publications. Due to the heterogeneity of summary measures and study designs, a quantitative synthesis or meta-analysis was not possible; consequently, a narrative synthesis was undertaken.

Studies were initially grouped into categories based on study type (i.e., observations and interventions). Interventions were allocated to sub-categories to allow for a well-structured narrative synthesis. Sub-categories included educational, labelling, financial, choice architecture, pre-existing guideline implementation and multi-component interventions.

## 3. Results

**Figure 1:**
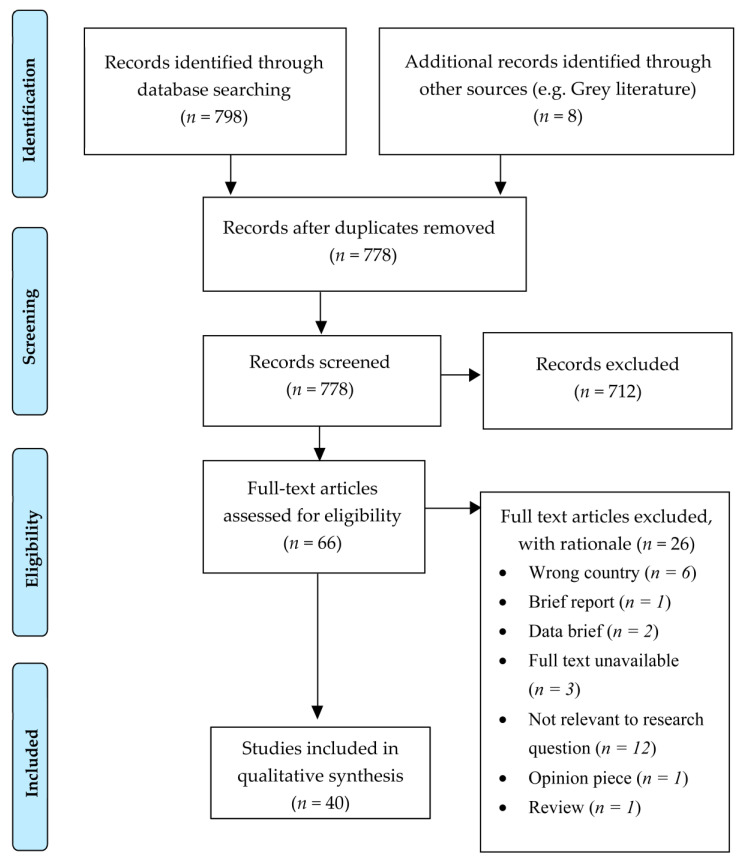
PRISMA 2009 flow diagram [15], detailing the number of studies included and excluded at each stage of the screening process.

Of the 806 search results initially identified from databases and search engines, 40 studies met the eligibility criteria. The most common reason for exclusion was the irrelevance of study outcomes to the research question. This was often due to a focus on patient meals rather than the food available to hospital employees and visitors. A PRISMA flow diagram [15] displays the inclusion and exclusion process (Figure 1).

### 3.1. Participant Characteristics

Fifteen publications reported the number of hospital employees, students or visitors involved in observations or interventions [16,17,18,19,20,21,22,23,24,25,26,27,28,29,30], while twelve reported the number of food outlets [31,32,33,34,35,36,37,38,39,40,41,42] and ten reported the number of healthcare facilities involved [43,44,45,46,47,48,49,50,51,52]. Three studies recorded the number of food outlets and survey respondents [53,54,55]. In total, the eligible publications reported the involvement of 18,171 participants, 139 food outlets and 529 hospitals and healthcare facilities.

### 3.2. Countries

In the UK, 5 interventions [31,34,38,39,40], 7 observations [17,23,35,37,44,45,51] and 1 mixed methods design [41] were reported; in the USA, 14 interventions [16,20,24,25,28,29,30,33,36,42,43,48,53,54,55], 10 observations [18,19,21,27,32,46,47,49,50,52] and 2 mixed methods studies [22,26] were reported.

### 3.3. Study Design

Observational studies (*n* = 17) employed a range of techniques, such as interviews, focus groups and cohort studies. Of the intervention studies (*n* = 20), 8 utilised a randomised controlled trial design [16,28,29,31,33,34,38,53], and 12 utilised quasi-experimental methods [20,24,25,30,36,39,40,42,43,48,54,55]; additionally, 3 studies employed mixed methods [22,26,41], incorporating a range of techniques, such as conducting interviews and collecting sales figures. The full data extraction can be seen in Table A1. According to the Quality Criteria Checklist for Primary Research, 11 of the eligible publications met high quality and risk of bias standards [16,18,26,27,28,29,31,38,48,49,53], while 29 were considered neither particularly strong nor particularly weak (Table A1) [17,19,20,21,22,23,24,25,30,32,33,34,35,36,37,39,40,41,42,43,44,45,46,47,50,51,52,54,55].

### 3.4. Observations

Five observational studies explored hospital food outlet adherence to pre-existing standards and guidelines [32,35,37,46,51]. Sustain (2017) found variation in the hospital food environments between 30 hospitals, with around 50% complying with standards listed in the NHS contract. Healthy options were also found to be more prevalent than unhealthy options in vending machines [51]. Similarly, James et al. (2017) investigated 30 food outlets across two NHS hospitals and their adherence to The National Institute for Health and Care Excellence (NICE) Quality Standard 94. Quality Standard 94 includes three quality statements relating to the availability of healthy options in vending machines (statement 1), nutritional information on menus (statement 2) and prominent display of healthy options (statement 3). Adherence to statements 1 and 2 was poor; only 10% of food products and 53% of drinks available in vending machines were classified as healthy and nutritional information was not available on menus at either hospital. Adherence to statement 3 was mixed, as both healthy and unhealthy options were displayed prominently in food outlets [35].

In 19 facilities across California, Lawrence et al. (2009) found that 81% of food in vending machines did not adhere to the California state nutrition standards for schools. Carbonated drinks were the most common beverages in vending machines, with advertisements for these beverages being prevalent. At the time of the study, 60% of facilities had already adopted or were beginning to implement nutritional standards for vending machines [46].

Mohinra et al. (2021) investigated the food environment in a dental hospital and found that beverages met Commissioning for Quality and Innovation (CQUIN) targets for sugar content; however, foods high in fat, salt and sugar were displayed in prominent locations, and unhealthy options were more affordable than healthy options [37].

Derrick et al. (2015) assessed the nutritional quality of cafeteria meals in relation to LiVe Well Plate guideline adherence. On average, food outlets that adhered to the LiVe Well Plate guideline had significantly higher nutrition composite scores than those which did not, particularly for point-of-purchase options, suggesting healthier food environments [32].

The diverse nutritional quality of cafeteria meals was also reported by Jaworowska et al. (2018). Variation was identified between different meals in the same outlet and between the same meals at different facilities; the majority of meals were high in saturated fat, while 69% of meat-based dishes and 43% of vegetarian dishes were high in salt content [44].

Findings were similar within paediatric hospitals or clinics. Across 14 facilities, Lesser et al. (2012) reported that most food outlets offered healthy options and half displayed nutritional information at the point of purchase. However, the majority had high-calorie options positioned close to point-of-purchase and promoting unhealthy options on signs was more common than promoting healthy options. Furthermore, half of the cafeterias had no healthy hot meals [47]. In vending machines accessible to children, Kibblewhite et al. (2010) found that none of the food-based or mixed food and drink vending machines contained 50% or more healthy food options. Meanwhile, 13% of drinks machines in paediatric clinics and 9% of drinks machines in other areas of the hospital contained 50% or more healthy options. Advertisements for brands associated with unhealthy products were also commonly found on vending machines [45].

Parental visitors to a paediatric hospital were broadly dissatisfied with the food environment. Food options were considered restrictive, and concerns were raised regarding the quality, freshness and positioning of products. Participants also felt that food available in the hospital food environment contradicted healthy eating messaging on signage [23].

Interviews, focus groups and surveys were also carried out with hospital employees and non-parental visitors. Bak et al. (2020) reported that nursing students believed that few healthy food options were available in hospitals. The students indicated that subsidising healthy foods could improve the hospital food environment and positively influence eating behaviours among nurses [17]. Barriers and facilitators to healthy eating were identified via interviews with 17 food service managers, carried out by Lederer et al. (2014). Only four of the respondents reported that their cafeteria followed nutrition standards set by the hospital (*n* = 3) or by the American Heart Association and the Academy of Nutrition and Dietetics (*n* = 1). The majority of respondents said that consumer-related factors, such as customer satisfaction and demand, were barriers to healthy food implementation [21]. Consumer satisfaction was also cited as a potential barrier by food service managers in a study by Jilcott Pitts et al. (2016). Other challenges included profit implications and training costs, while potential facilitators of healthy eating included altered positioning of healthy and unhealthy options and signage promoting healthy options [19]. Furthermore, Liebert et al. (2013) also highlighted profits and resources as potential barriers to implementing nutrition interventions. Despite this, over 80% of respondents were concerned about eating well and stated that they would be more likely to do so if healthy options were cheaper than unhealthy options. Additionally, 73% were in favour of the taxation and subsidisation of products based on nutritional content [22].

The impact of hospital location or average visitor socioeconomic status on the hospital food environment was also explored. Winston et al. (2013) found no significant relationship between the socioeconomic status of the local area and the nutrition composite score of the hospital [52]. In contrast, a study by Goldstein et al. (2014) found that physicians seeing mostly patients of higher socioeconomic status were more likely to report high levels of nutritional support compared to those seeing patients of lower socioeconomic status [18].

### 3.5. Interventions

Hospital food environment interventions can be grouped into several categories. Studies incorporating interventions from three or more categories are classified as “multi-component”; these studies are grouped and explored separately under the “Multi-Component Interventions” subheading.

#### 3.5.1. Educational

Educational interventions aim to increase consumer knowledge of the nutritional guidelines or the nutritional content of foods. Of the seven educational interventions, four utilised signage or flyers to increase awareness of the nutritional content of products.

Allan and Powel (2020) assessed the impact of point-of-purchase signage on the nutritional quality of purchases and found that signage reduced the calorie content of purchases, reduced sugar content in some circumstances and had no impact on fat content [31]. Webb et al. (2011) also introduced nutritional labelling on posters or nutritional labelling on posters and a point-of-purchase menu board. More consumers noticed the nutritional information when posters and menu boards were used, compared to only posters. Nutritional labelling on posters and menu boards was also associated with the increased purchase of lower-calorie snacks and side dishes but made no significant difference in the nutritional content of entrée purchases [55].

When combining signage with traffic light labelling, Sonnenberg et al. (2013) found that nutritional content, taste and price became more important to consumers, while convenience became less important. Participants who were influenced by nutritional information bought more healthy products than those who were not [25]. Block et al. (2010) introduced signage and flyers about the health implications of regular soft-drink consumption, along with taxation. Education alone had no significant effect on sugar-sweetened soft drink sales. However, education enhanced the effects of taxation, reducing soft drink sales by 10% compared to price increases alone [53].

Two studies used digital methods to deliver nutritional information [16,28]. Abel et al. (2015) sent texts or emails to participants regarding calorie reference values. Participants who received the information were twice as likely to know reference values compared to a control group, but this did not appear to alter calorie consumption or portion sizes [16]. Thorndike et al. (2021) used emails (and letters) to provide feedback on food choices. The number of healthy purchases increased while the number of unhealthy purchases decreased. These effects remained significant at a 24-month follow-up, but there was no significant change in weight status [28].

Another study by Thorndike et al. (2016) used social norm feedback in combination with financial incentives. Social norm feedback alone led to a 1.8% increase in healthy purchases, but this was not statistically significant. Combining social norm feedback with a financial incentive resulted in a 2.2% increase in healthy purchases, and employees rated healthiest at baseline were influenced most greatly by the interventions [29].

#### 3.5.2. Labelling

Six studies explored the effects of labelling [25,27,30,33,42,54]. Elbel et al. (2013) added ‘less healthy’ labels to some items, and this increased purchase of healthier options by 7% [33]. Sato et al. (2013) added calorie, fat and sodium content information to packaging. A non-significant increase in healthier options sold was observed, along with a significant decrease in the total number of meals sold per day. Despite this, 71% of customers who noticed the intervention reacted positively to it, and 50% claimed that the labels influenced them to purchase a healthier option [54].

The most common form of labelling was traffic light labelling. Sonnenberg et al. (2013) investigated the impacts of traffic light labelling and nutritional signage on customer food-related attitudes. The intervention increased the importance of health and nutrition to participants, and more participants claimed to use nutritional information when making food choices during the intervention compared to pre-intervention. More healthy options were purchased by those who were influenced by the labels than those who were not [25].

Traffic light labelling reduced the number of unhealthy purchases in three studies. Whitt et al. (2018) assessed the impact of traffic light labelling on food choices compared to cartoon labelling. Traffic light labelling decreased unhealthy food purchases by 7% from baseline, while cartoon labelling increased the number of unhealthy purchases by 1% from baseline and by 5% from the washout period [42]. In two studies, Thorndike et al. (2014, 2019) found that traffic light labelling decreased the proportion of red-labelled products purchased [30], which resulted in fewer calories purchased and potential employee weight loss [27].

#### 3.5.3. Financial

The effects of financial interventions were primarily investigated via taxation and subsidisation of products. Elbel et al. (2013) found that taxing unhealthy products increased the proportion of healthy purchases by 11.5% from baseline and that this was associated with fewer unhealthy purchases and an increased proportion of healthy beverage purchases [33]. Similarly, Block et al. (2010) found that increasing the prices of sugar-sweetened soft drinks decreased sales by 26% [53]. Patsch et al. (2016) utilised taxation and subsidisation and found significant increases in the proportion of healthy alternatives sold along with decreases in the number of traditional, less healthy products sold [24].

In addition to feedback-based interventions, Thorndike et al. (2016) offered financial incentives for healthy purchases. Feedback plus a financial incentive led to a 2.2% increase in healthy purchases, compared to a 1.8% increase for feedback alone and a 0.1% increase for the control group [29]. In a second study by Thorndike et al. (2021), the intervention increased the purchase of healthy products by 7.3% and decreased the purchase of unhealthy options by 3.9%. This effect did not lead to significant weight loss in the intervention group [28].

#### 3.5.4. Choice Architecture

Choice architecture was implemented in several ways; the most prevalent method was altering the proportion of healthy and unhealthy products available to purchase. Three studies focused on products available in vending machines. Griffiths et al. (2020), Grivois-Shah et al. (2018) and Pechey et al. (2019) found an increase in the number of healthy products purchased [43], a decrease in the amount of calories purchased [34,38] and mixed results regarding the financial impact of the intervention [34,43]. Simpson et al. (2018) conducted a similar study in a hospital shop but found no significant difference in the relative proportion of healthy options sold between pre- and post-intervention sales data [40].

Thorndike et al. (2014) took a different approach and utilised choice architecture by making healthy options more visible. The impact of choice architecture itself is unknown, as it was only assessed in combination with traffic light labelling. Nevertheless, the overall intervention decreased the proportion of unhealthy product sales by 3% and increased the proportion of healthy product sales by 5% after two years [30].

Public Health England, as previously known (2018), investigated the impact of altering product positioning in vending machines; this is explored in more depth in the ‘Implementing Standards and Guidelines’ section [39].

#### 3.5.5. Implementing Standards and Guidelines

Three studies assessed the impacts of supporting the implementation of pre-existing standards for hospital food outlets [39,41,48]. Moran et al. (2016) encouraged the implementation of the Healthy Hospitals Food Initiative and improved adherence to the programme. The nutritional quality of the hospital food environment also improved [48].

Stead et al. (2020) focused on the implementation of Healthcare Retail Standards in hospital shops. Compliance with these standards had no effect on the number of fruit products available but decreased the number of chocolate-based options and the number of promotions for these products. The standards also reduced meal-deal sales [41].

Public Health England (2018) altered the content of vending machines in line with Government Buying Standards for Food and Catering. In drinks machines, these changes decreased calorie and sugar content of purchases and increased proportion of ‘diet’ beverages sold. In food machines, sales of crisps decreased while sales of confectionary and dried fruit and nut products increased [39].

#### 3.5.6. Multi-Component Interventions

Stites et al. (2015) carried out a study involving choice architecture, financial incentives and educational components. Hospital employees were taught mindfulness techniques, encouraged to pre-order meals and, for part of the intervention, provided with vouchers for the cafeteria. This intervention resulted in lower calorie and fat purchases compared to a control group, with and without the vouchers. Despite the dietary changes, weight loss was not significant among the intervention group [26].

LaCaille et al. (2016) incorporated signage, traffic light labelling and choice architecture into a nutrition intervention, alongside encouraging physical activity participation. The control group experienced a greater average reduction in waist circumference than the intervention group after six months (but not after 12 months). As well as this, the intervention group experienced a significant decrease in fruit, vegetable and fibre intake over the course of the study. Consumption of foods high in sugar and fat also decreased [20].

Mazza et al. (2017) also reported mixed results. Financial interventions and traffic light labelling were combined with health and social norm messaging and choice architecture. Financial interventions and traffic light labelling increased healthy beverage purchases by 2.9%. The addition of health and social norm messaging and grouping items into nutritional categories further increased healthy beverage purchases. Healthy crisp sales increased by 5.4% when traffic light labelling was introduced and by 6% when health messaging was implemented. However, healthy crisp sales decreased by 5.9% when the price of water was reduced, suggesting that this financial intervention nullified the beneficial effects of traffic light labelling [36].

## 4. Discussion

The observational studies carried out across the UK and USA suggest that the quality of the wider food environment is diverse. Compliance with pre-existing standards and guidelines is varied [32,35,37,46,51] and the nutritional quality of cafeteria meals differs between meals and facilities [44,47]. A lack of healthy food options was reported in vending machines, while the availability of healthy beverage options was slightly greater [45]. Hospital visitors and employees reported concerns regarding the quality, freshness and positioning of healthy and unhealthy options [23] and believed there was a lack of healthy options available [17]. Barriers to the implementation of healthy eating initiatives were also identified, including customer satisfaction [19,21] and profit implications [19,22], although participants were in favour of a financial intervention to encourage healthy food and beverage choices [22]. Findings relating to the impact of socioeconomic status on the hospital food environment are inconsistent [18,52].

Utilising signage and flyers is associated with the reduced calorie content of purchases [31], and displaying nutritional information on menu boards and posters increases the purchase of low-calorie options compared with using posters alone [55]. Digital methods of communicating nutritional information can increase knowledge of reference values [16] and increase purchase frequency of healthy options whilst decreasing purchase frequency of unhealthy options [28], but effects on calorie consumption are contested [16,28] and these interventions have no significant impact on weight-related outcomes [28]. Moreover, educational interventions can be successfully incorporated with traffic light labelling [25] and financial interventions [29,53].

Adding simple labels to products, marking them as ‘less healthy’ or giving some nutritional information, is associated with an increase in the number of healthy purchases [33] but also with decreased total purchases per day [54]. Nevertheless, labelling interventions are viewed positively by consumers [54]. Traffic light labelling has been shown to increase the importance of nutrition to participants [25], increase the number of healthy purchases [25], reduce the number of unhealthy purchases [30,42] and reduce the calorie content of purchases [27]. It was predicted that this could lead to consumer weight loss, provided that no other lifestyle alterations occurred [27].

Taxation on unhealthy products or subsidisation of healthy products was found to be associated with an increased proportion of healthy purchases [24,33] and a decreased number of sugar-sweetened soft drink purchases [53]. Financial incentives were also found to effectively increase healthy purchases and decrease unhealthy purchases [28,29], but no impact on weight-related outcomes was identified [28].

Altering the proportion of healthy options available to purchase from vending machines was found to increase healthy purchases [43] and decrease the calorific content of purchases [34,38]. This type of intervention may have undesirable financial outcomes for food outlets, but this remains unclear [34,43].

One study found no change in the proportion of healthy options sold before and after a choice architecture intervention in a hospital shop [40]. However, another study reported that displaying healthy options more prominently reduced unhealthy purchases and increased healthy purchases when combined with traffic light labelling [30]. Choice architecture interventions also increased ‘diet’ beverage sales and reduced the total sugar content of purchases [39].

Encouraging implementation of pre-existing standards and guidelines is associated with an overall improvement in the hospital food environment [48] and decreased availability of unhealthy products [41]. In beverage vending machines, implementing governmental standards increased the proportion of ‘diet’ beverage sales and reduced the sugar and calorie content of purchases [39]. However, adherence to these standards has also been shown to reduce sales of meal deals [41] and increase sales of confectionary [39].

Multi-component interventions have been carried out with a range of study designs. These interventions have been shown to reduce calorie and fat content of purchases [26], reduce consumption of foods high in sugar and fat [20], increase healthy beverage purchases [36] and increase the purchase of healthy snack options [36]. However, certain multi-component interventions have also resulted in decreased fruit, vegetable and fibre intake [20] and decreased sales of healthy snack options [36]. These interventions were not associated with significant weight loss [20,26].

This review has several strengths. Study screening was independently conducted by two researchers, and the process was blinded to reduce the impact of researcher bias. Additionally, conducting a narrative synthesis allowed the integration of material that would have been incomparable using quantitative synthesis. Therefore, the heterogeneous data have been compiled into a useful summary to inform further research. However, the heterogeneity of study designs and outcome measures restricts the use of quantitative synthesis and meta-analysis to summarise findings. Additionally, many studies involve multiple interventions, making it difficult to determine the impact of each intervention on outcomes. This limits the strength of recommendations.

Another limitation of this review is our pragmatic decision to restrict the search to studies carried out in the UK and USA. This decision was taken because the number of studies carried out in the UK and USA allowed for an in-depth analysis of all relevant literature within the available time frame. It should be noted that the exclusion of studies from other countries limits the wider generalisability of findings.

Furthermore, some studies took place several years ago, meaning that information about the ‘current hospital food environment’ may no longer be valid. One observation found that 60% of facilities were beginning to adopt vending machine nutrition standards in 2009 [46], so vending machine nutritional quality could have since changed. Nevertheless, poor nutritional quality in vending machines was reported in 2017 [35], indicating that concerns about healthy vending machine options may remain relevant.

The majority of interventions discussed in this review can be used to improve nutrition awareness, eating behaviour or the overall hospital food environment. Studies that surveyed hospital employees or visitors on their acceptance of these interventions reported that over 70% of responses were positive [22,54].

Some studies show that combining multiple interventions can improve the nutritional quality of food purchases. However, multi-component interventions may have the potential to lead to detrimental impacts, such as reduced fruit and vegetable consumption and reduced sales of healthy snacks [20,36]. Consequently, more robust study designs are required to identify the most effective intervention combinations in multi-component studies.

Further interventions are needed in the UK to investigate the most effective methods of improving the nutritional quality of employee and visitor diets. Research into the associations between food environment and food intake in a variety of settings, other than hospitals, would also be valuable. Moreover, some interventions included in this review would benefit from being replicated to generate an evidence base with consistent outcome measures. This would produce more homogenous data, facilitating quantitative synthesis and meta-analysis. A more precise estimated effect size would be generated, thereby strengthening practice and policy recommendations.

Timely implementation of public health interventions, such as altering food environments and encouraging healthier diets, is especially pertinent in the wake of the COVID-19 pandemic. Dietary patterns high in fat, salt and sugar contribute to the prevalence of obesity and type II diabetes, which increase the risk of severe COVID-19 outcomes [56]. By altering the hospital food environment, healthy food and beverages could be made the easiest option to purchase, thereby improving dietary quality and potentially reducing the risk of ill-health among hospital employees and visitors. Food environment interventions could also reduce the discrepancy between health messaging and poor hospital food environments, ensuring that hospitals act as positive role models for healthy lifestyle behaviours.

## 5. Conclusions

In conclusion, the quality of the hospital food environment varies within and between facilities. Hospital visitors and employees are generally receptive to food environment interventions and a variety of designs can be used to improve the hospital food environment and increase the proportion of healthy purchases. However, multi-component interventions can have neutral or detrimental effects on participant eating behaviours depending on the design. Therefore, further research that also encompasses studies beyond the UK and USA is required to determine the most effective combinations within multi-component interventions.

## Data Availability

Not applicable.

## Appendix A

### Appendix A.1. Scopus Search Criteria

Hospital AND (food OR nutrition*) AND (“convenience food” OR “fast food” OR “healthy food” OR snack OR fat OR salt OR sugar OR calorie OR beverage) AND (catering OR outlet OR vending AND machine OR cafe* OR restaurant OR canteen OR “hospital shop” OR “hospital store” OR “gift shop”) AND (policy OR “food preference” OR “consumer attitudes” OR choice OR decision OR “health promotion” OR diet OR healthy OR options) AND (LIMIT-TO (AFFILCOUNTRY, “United States”) OR LIMIT-TO (AFFILCOUNTRY, “United Kingdom”)) AND (LIMIT-TO (LANGUAGE, “English”)).

### Appendix A.2. Embase, Medline and APA PsycInfo Controlled Vocabulary Search

Hospital AND (Food OR Food product OR Convenience food OR Packaged food OR Fat OR Dietary fat OR Fat intake OR Dietary fat OR Sodium chloride OR Salt intake OR Dietary salt OR Sugar OR Dietary sugar OR Sugar intake OR Beverage OR Sugar-sweetened beverage OR Calorie) AND (Catering service OR Food outlet OR Food service OR Vending machine OR Automatic food dispensers OR Commercial food OR Cafeteria OR Cafeteria diet OR Restaurant OR Canteen OR Shop OR Store OR Retail OR Commerce) AND (Nutrition policy OR Healthcare policy OR Government policy making OR Public health OR Healthcare planning OR Health policy OR Nutrition OR Nutritional value OR Nutritive value OR Food preference OR Feeding behaviour OR Consumer attitudes OR Consumer satisfaction OR Eating behaviour OR Choice OR Decision making OR Health promotion OR Food availability OR Diet OR Healthy diet OR Food options).

## Appendix B

**Table A1:** Full data extraction for *n* = 40 studies, including quality assessment and risk of bias analysis, carried out using the Quality Criteria Checklist for Primary Research [14].

Citation, Country	Study Design, Duration	Aim of Study	Participants/ Hospitals	Intervention	Outcome Measures	Results	Risk of Bias Analysis
Abel et al. (2015) [16], USA	RCT, 4 weeks	Assess knowledge of government reference values among the public and assess the impact of email/text interventions on calorie reference value knowledge.	*n* = 246 hospital employees and students	Assess knowledge of reference values, deliver weekly text or email prompts regarding calorie intake, then administer a follow-up test to assess impact of intervention.	Knowledge of government reference values and impact of intervention on self-reported calorie consumption.	At baseline, 42.2% of participants knew the 2000 calorie reference value. Following text intervention, participants 2x as likely to know the reference value as the control group (*p* = 0.047, odds ratio = 2.2, 95% confidence interval [1.01, 4.73]). No significant difference between text and email conditions. 52% of participants would use the information when making future food decisions. 32% stated that the intervention led to lower calorie intake than if the information was available on menus and posters—no statistically significant change in self-reported calorie consumption or portion size.	+
Allan and Powell (2020) [31], UK	RCT, 6 months	Reduce purchase of unhealthy single-serve snacks.	*n =* 30 hospital food outlets	Implement tailored point-of-purchase signs displaying calorific values of the items for sale.	Average energy, fat and sugar content of purchases per day, average cost of each purchase and total number of purchases per day.	Purchases significantly lower in calories (95% CI: −0.83, −2.85, *p* < 0.001), sugar content and cost (95% CI: −0.46, −1.32, *p* < 0.001) post-intervention compared to pre-intervention. This was also true for calories (*p* = 0.049) and cost (*p* = 0.03) comparing intervention to the control site. No significant differences in fat content (*p* = 0.07), sugar content (*p* = 0.48) or number of purchases (*p* = 0.64) between intervention and control sites.	+
Bak et al. (2020) [17], UK	Observational, 2 h (per focus group)	Investigate beliefs of student nurses about causes of nurses’ health-related behaviours, plus strategies to improve these behaviours.	*n =* 20 undergraduate nursing students	Ask student nurses about underlying factors for health-related behaviours, reasoning behind these behaviours and identifying stakeholders responsible for implementing solutions.	Student views regarding the underlying causes of health-related behaviours among nurses and how these could be improved.	Four key causes of negative health-related behaviours identified: Knowledge, shift-work, culture and stress. Several students reported snacking was common during night-shifts and few healthy food options were available within hospitals. They also suggested that high stress triggers a desire to eat “comfort foods”, which are often high in fat. The idea of subsidising healthy food options for staff was raised as a possible strategy to improve food-related behaviours.	∅
Block et al. (2010) [53], USA	RCT, 6 weeks	Assess the impact of increasing the prices of sugar-sweetened soft drinks and educational interventions on beverage sales.	*n* = 1 hospital food outlet and *n =* 154 survey respondents	5 phase intervention involving 35% price increase on soft drinks, an educational campaign (posters and flyers) and combined price increase and educational campaign.	Number and category of drinks purchased per day and total number of beverage sales.	Sales of regular, sugar-sweetened soft drinks significantly decreased during intervention, while sales of diet soft drinks increased. Regular soft drink sales decreased by 26% during price increase (95% CI = 39.0, 14.0) and 36% (95% CI = 49.0, 23.0)in the combination phase (education and price increase). Education alone did not significantly impact sales of regular soft drinks, despite a 9% sales increase (95% CI = −4.0, 22.0). 44% of survey participants noticed an intervention, with 82% being aware of the educational phase and 18% being aware of the price increase.	+
Derrick et al. (2015) [32], USA	Observational, short duration	Describe nutrition environments in hospitals that participate in the One Health Care System and investigate the impact of the LiVe Well Plate initiative.	*n =* 21 hospital food outlets	Assess food environment using the Hospital Nutrition Environment Scan, including signage, menu information and pricing strategies. Implement the LiVe Well Plate in low-scoring hospitals based on menu factors.	Nutrition composite scores of cafeterias and nutrition scores based on barriers and facilitators, grab-and-go items, menu offering and point-of-purchase options.	Mean nutrition composite score was 49.2 ± 8.1 in hospitals which adhered to the LiVe Well Plate and 29.7 ± 11.3 in hospitals which did not. Those adhering to the initiative had significantly higher scores for facilitators and barriers (*p* < 0.001) and point-of-purchase options (*p* = 0.013) than the other group and two locations promoted healthy food choices via pricing. No significant differences between groups for grab-and-go (*p* = 0.178) or menu options (*p* = 0.172).	∅
Elbel et al. (2013) [33], USA	RCT, 6 months	Investigate the impact of taxation and food labelling interventions on healthy produce purchases.	*n =* 1 hospital food outlet	5 phase intervention involving product labelling, a 30% taxation on less healthy items, a combined intervention and a combined intervention with taxation rationale stated on products.	Number and nutritional quality of purchases.	At baseline, 47.2% of purchases were healthy (95% CI = 43%, 52%). This rose to 54% in the labelling condition (95% CI = 49%, 58%) and 59% under the taxation conditions (95% CI = 56%, 61%; not shown). Taxation conditions did not significantly differ (*p* on Wald test = 0.82); all increased probability of healthy purchase by 10–12% (*p* < 0.001). Taxation associated with fewer unhealthy food choices (AME = −9.41%, 95% CI = −13.80%, −5.03%, *p* < 0.001) and more healthy beverage purchases (AME = 5.87%, 95% CI = 2.36%, 9.38%, *p* = 0.001). Unclear if taxation had a greater impact than labelling.	∅
Goldstein et al. (2014) [18], USA	Observational, short duration	Investigate physician perspectives on hospital support available to promote healthy hospital environments.	*n =* 1485 physicians	Physicians were asked to rate their place of work based on support offered for achievement and maintenance of a healthy food environment and physical activity.	Physician rating of hospital support	Health-promoting environments were mostly rated ‘good’, with 70% of respondents suggesting that nutrition environments were supportive. Responses varied according to socioeconomic status of the average patient (higher ratings were given by physicians seeing lower middle class [OR: 1.74 (1.27–2.39)], upper middle class [2.23 (1.61–3.09)] to affluent patients [2.91 (95% CI: 1.49–5.66)] compared to physicians seeing very poor to lower/middle-class patients). 40% of respondents stated that their facilities supported healthy nutrition.	+
Griffiths et al. (2020) [34], UK	RCT, 24 weeks	Assess health benefits and cost-effectiveness of replacing regular snacks with healthy options.	*n* = 2 hospital food outlets	Vending machines in 2 locations were exposed to alternating “healthy” or “unhealthy” conditions, with all products costing the same amount.	Sales volume, profit and calories sold. Compensatory behaviours (sales data from nearby shop), number of items purchased by each customer and time taken to complete each purchase	The healthy condition was associated with a 61% decrease in calories purchased, which was significant (SE = 579.23; t = −3.868; *p* < 0.0001) and a GBP 1116 decrease in profits. There was no significant impact on number of sales and no significant association between calorie content and sales volume. No significant difference in sales from a local shop (SE = 0.848; t = 0.249; *p* = 0.81), suggesting no compensatory behaviours. No significant difference in the likelihood of single versus multiple item purchases between conditions (χ^2^ (1) = 2.20, *p* = 0.14).	∅
Grivois-Shah et al. (2018) [43], USA	Quasi-Experimental, 6 months	Assess the impact of increasing the proportion of healthy options in vending machines on calorie, fat, sugar and salt purchase, plus sales revenue.	*n =* 23 hospitals	Proportion of healthy “Right Choice” items in vending machines was increased from 20% to 80%.	Percentage of healthy options vended and total number of items vended. Mean revenue, calorific value, fat content and sodium content per site, per month.	The intervention increased average number of “Right Choice” items purchased from vending machines from 9.9% to 35%. Percentage change in average monthly revenue was not significantly different between baseline and post-intervention (95% CI = −12.6 to 7.8, *p* = 0.5766). On average, the intervention reduced average fat content by 27.4% per month (95% CI = −37.4 to −15.9, *p* < 0.0001), sugar content by 11.8% per month (95% CI = −22.0 to −0.3, *p* = 0.0447), sodium content by 25.9% per month (95% CI = −33.9 to 17.0, *p* < 0.0001) and calorie content by 16.7% per month (95% CI = −25.5 to −6.8, *p* = 0.0016). Beverage profit declined by 11.1% (95% CI = −19.9 to −1.3, *p* = 0.0274) while number sold increased by 16.2% (95% CI = 3.7 to 30.2, *p* = 0.0100).	∅
James et al. (2017) [35], UK	Observational, 2 weeks	Assess adherence to NICE quality statements 1–3 of quality standard 94 at two NHS hospitals.	*n =* 30 hospital food outlets	Food environments were assessed using the Consumer Nutrition Environment Tool. Adherence to quality statements was measured. Statement 1 regarding healthy options in vending machines, statement 2 about nutritional information on menus and statement 3 regarding prominent display of healthy options.	Proportion of healthy and less healthy options in vending machines, clarity of nutrition information on menus and prominence of healthy food and beverages displayed.	10% of food products and 53% of drinks in vending machines were considered healthy, making adherence to quality statement 1 poor. Food items were given a C-NET score of 18.3. Nutritional information was not available on menus at either facility, so adherence to quality statement 2 was also poor. Adherence to quality statement 3 was inconsistent, as both healthy and less healthy products were prominently displayed in cafeterias. 25% of cafeteria options were healthy.	∅
Jaworowska et al. (2018) [44], UK	Observational, 2 months	Describe nutritional quality of hot lunches in NHS hospital staff canteens.	*n =* 8 hospitals	Nutritional composition of canteen meals was assessed using meal samples from each canteen.	Energy, protein, total fat, saturated fat, carbohydrates, fibre and sodium content of meals.	Meals containing meat had a higher energy density than vegetarian meals and were also higher in salt content (0.61 vs. 0.49 g; *p* < 0.05) and protein per 100g (9.8 vs. 4.8 g; *p* < 0.05). Significant variation in nutritional composition between different meals. According to standard cafeteria portion sizes, 67% of meat-based and 80% of vegetarian meals were high in saturated fat, while 69% of meat-based and 43% of vegetarian dishes were high in salt (red light according to the traffic light labelling system). Meals varied significantly between hospitals, especially per portion.	∅
Jilcott Pitts et al. (2016) [19], USA	Observational, short duration	Describe barriers and facilitators to the implementation of healthy service guidelines and strategies for health promotion.	*n =* 9 food service managers and operators	Information about hospital size, types of food available and current nutrition initiatives was gathered via a quantitative survey and more in-depth information was obtained from qualitative interviews.	Difficulty or ease of guideline implementation, price outcomes, barriers and facilitators to implementation and potential behavioural design strategies to promote healthy eating.	Challenges raised regarding implementation of guidelines including profit implications, customer dissatisfaction and difficulties with changing obligations to food strategies. Suggested strategies to encourage healthier choices included signage and icons on healthier items, positioning of healthy options and marketing techniques. Additional training costs were anticipated to arise from altering the food environment.	∅
Kibblewhite et al. (2010) [45], UK	Observational, short duration	Describe products available in vending machines close to paediatric wards and outpatient clinics.	*n =* 13 hospitals	Percentages of healthy and unhealthy vending machine items accessible to children were calculated.	Number of healthy and unhealthy items in vending machines and advertising for unhealthy brands.	In paediatric clinics, 13% of the drinks-only machines contained over 50% healthy options, while none of the food-only machines reached this target. The mixed food and drink machine met the target for drinks, but not food. In other areas of the hospital which were accessible to children, 9% of drinks machines contained over 50% healthy options compared to 27% for food machines. Mixed machines contained 50% healthy drinks but not food. 55% of machines in paediatric clinics and 72% in other areas displayed commercial logos, mostly associated with unhealthy products.	∅
LaCaille et al. (2016) [20], USA	Quasi-Experimental, 12 months	Assess the efficacy of *Go!*, an obesity-prevention programme.	*n =* 900 hospital and primary care clinic employees	A multi-component intervention was implemented, including traffic light labelling, choice architecture, pedometer usage and signage was launched.	Weight, BMI, waist circumference, physical activity levels and dietary behaviour after 6 months and 1 year	No significant change in weight (95% CI = −1.13, 1.56, *p* = 0.76) or BMI between groups (95% CI = −0.17, 2.39, *p* = 0.09) or in weight (95% CI = −0.36, 0.89, *p =* 0.40) or BMI (95% CI = −0.27, 0.95, *p* = 0.27) over time. The control group had a significantly greater decrease in waist circumference than the intervention group at 6 months (95% CI = 1.28 to 4.72, *p* = 0.001) but not at 12 months (95% CI = −1.06, 2.16, *p =* 0.51). There was a significant decrease in fruit and vegetable intake over 12 months in the intervention group (95% CI = −13.13, −2.23, *p* = 0.007), but consumption of foods high sugar and fat, like cookies, cakes and brownies, also significantly decreased (95% CI = −0.12, −0.01, *p* = 0.02). The intervention caused employees to view their employer as more committed to improving health and wellbeing (95% CI = 0.06, 0.23, *p* = 0.002) and 86% wanted the intervention to continue.	∅
Lawrence et al. (2009) [46], USA	Observational, short duration	Describe the range of healthy and unhealthy vending machine options and healthcare settings.	*n =* 19 healthcare facilities	Numbers of healthy and unhealthy products in vending machines were recorded and the quality and quantity of food products were compared between 3 types of environment.	Percentage of healthy options in vending machines, advertising and implementation of standards.	In hospitals and clinics, carbonated beverages were the most prevalent drink, accounting for 30% of drinks in hospitals and 38% in clinics). 81% of food across all sites did not adhere to standards, 75% of vending machines displayed advertisements for carbonated beverages and 60% of facilities with vending machines were in the process of adopting nutritional standards for the machines.	∅
Lederer et al. (2014) [21], USA	Observational, short duration	Describe the nutritional knowledge, practices and attitudes of hospital cafeteria managers in hospitals following the Healthy Hospitals Food Initiative (HHFI).	*n =* 17 cafeteria managers	A 22 question survey was delivered to participants who approved menus, had influence over food purchases and monitored food preparation.	Nutritional practices, standards and policies.	4 of 17 participants said that their cafeteria followed hospital nutrition standards. 13 claimed to think about nutrition when planning menus, but most respondents ranked consumer preferences and cost as the 2 main considerations. 14 participants reported reducing sodium content of meals by cooking from scratch, buying products with lower sodium content and decreasing the salt content of recipes. 16 respondents cited consumer-related factors as limitations to healthy food implementation, such as lack of demand, customer satisfaction and lack of consumer education around healthy eating. Environmental factors were also a concern for 6 participants, such as an inability to move cafeteria fixtures to make healthy options more prominent.	∅
Lesser et al. (2012) [47], USA	Observational, short duration	Describe the quality of the food environments in outlets at a children’s hospital	*n =* 14 hospitals	The Nutrition Environment Measures Study in Restaurants was adapted for use in cafeterias. Items in hospital cafeterias were scored according to healthy or unhealthy status.	Nutritional quality of food in hospital cafeterias and healthy eating prompts.	Majority of venues offered healthy options, like low-fat milk, fresh fruit and a salad bar. Around 50% of cafeterias displayed point-of-purchase nutritional information, while less than 33% displayed signage promoting healthy menu choices. High-calorie options were available near point-of-purchase in 81% of venues, 50% offered discounts for multiple purchases, 38% displayed signage promoting unhealthy eating and 50% had no healthy hot meals. NEMS-C scores ranged from 13 to 30, with a mean of 19.1.	∅
Liebert et al. (2013) [22], USA	Mixed Methods, 2 years	Research and plan the Better Bites intervention programme to improve food choices of hospital employees.	*n =* 100 hospital employees	Employees were interviewed using the Nutrition Environment Measures Study in Restaurants and other surveys. Best practices were identified for planning and developing the Better Bites intervention.	Barriers and facilitators to healthy eating, perceptions of healthy food availability and likelihood of behaviour modification following interventions.	Majority of respondents supported the intervention but concerns were raised about profits, resources and ability to change eating behaviours. 82% of respondents were concerned about eating well and 83% reported being more likely to buy healthy items if cheaper than unhealthy items. 73% in favour of reducing healthy option prices and increasing unhealthy option prices.	∅
Mazza et al. (2017) [36], USA	Quasi-Experimental, short duration (around 15 days per intervention)	Comparing the impacts of a range of interventions, combined with traffic light labelling, on beverage and crisp (potato chip) sales.	*n* = 1 hospital food outlet	Interventions, such as price increases, health messaging, social norm messaging and grouping items into nutritional categories, were assessed for efficacy alongside a traffic light labelling intervention.	Daily number of healthy (green-labelled) purchases.	Traffic light labelling increased healthy beverage purchases by 2.9% compared to a price increase alone (*p* < 0.0001). When labelling and price changes were combined with subsequent interventions, colour grouping, social norm feedback and oppositional pairing reduced healthy beverage purchases by 2% (*p* < 0.0001), 1.7% (*p* < 0.01) and 6.9% (*p* = 0.01), respectively. For crisps, traffic light labelling increased percentage of healthy crisps sold by 5.4% (*p* = 0.001), compared to a sugar sweetened beverage price increase. When water price decreased, healthy crisp sales decreased by 5.9% (*p* = 0.003). Health messaging increased healthy crisp sales by 6% compared to control conditions (*p* = 0.004).	∅
McSweeney et al. (2018) [23], UK	Observational, short duration	Assess parental perspectives of food available in a children’s hospital and the barriers and facilitators to healthy eating.	*n =* 18 parents	Parents were interviewed regarding ease of healthy eating in the hospitals until no new themes were raised.	Themes centred around food accessibility and nutritional quality of food in hospitals.	Purchases were influenced by cost and speed. Parents described the food choice as restrictive, especially for children, vegetarians and those trying to eat healthier. Quality of food was said to vary between outlets and concerns about freshness, presentation and location of unhealthy options were raised. Maintenance of a healthy diet was considered difficult, as food available contradicted healthy eating messages shown on signs. A discount or loyalty card was proposed to make healthy food cheaper to repeat visitors.	∅
Mohindra et al. (2021) [37], UK	Observational, 2 weeks	Describe nutritional quality of products available in a dental hospital, along with the price and positioning of high fat, salt and sugar products.	*n =* 1 hospital food outlet	An audit of coffee shop food and beverage options was carried out using Commissioning for Quality and Innovation (CQUIN) indicator 1b targets for 2018/19	Nutritional content of packaged food and drinks and fresh food, total sugar content in products and the price, quantity and variety of each product category.	A variety of pre-packaged sandwiches, wraps and salads was available, compared to just 1 packaged vegetable product, 3 packaged fruit products and 3 fresh fruit products. 42% of packaged sandwiches, wraps and salads contained less than 400 kcal and below 5 g saturated fat per 100 g. 50% of cakes and 66% of biscuits adhered to the CQUIN guideline of containing less than 250 kcal per portion and 12% of cakes contained more sugar per portion than the daily recommended sugar intake from SACN. All crisps and popcorn met targets for saturated fat, while 73% contained less than the PHE salt target. All cold drinks met the CQUIN targets per 100 mL, but portion sizes varied widely and, as such, so did sugar content per portion. All hot drinks met the CQUIN targets. Unhealthy foods were displayed prominently compared to fresh fruit and packaged fruit was more expensive than packaged biscuits. Low-fat sandwiches were also more expensive than high-fat sandwiches.	∅
Moran et al. (2016) [48], USA	Quasi-Experimental, 3 years	Implement the Healthy Hospitals Food Initiative (HHFI) in public and private hospitals to establish nutrition standards and assess outcomes.	*n =* 40 hospitals	HHFI implementation was supported by dieticians, promotional materials and monthly progress reports to identify achievements and next steps.	Degree of HHFI implementation and nutritional quality of food options.	At baseline, all public hospitals (*n =* 16) had implemented standards for patient meals and vending machines, but none had implemented cafeteria standards. No hospital met the criteria for sodium or whole grains, and none offered an affordable healthy meal. Following intervention, 12 public hospitals met cafeteria standards, while 71% of private hospitals had implemented standards for patient meals, 58% for beverage vending machines, 50% for food vending machines and 67% for cafeterias. 21% of hospitals achieved sodium standards, 61% achieved standards for whole grains and 68% offered a healthy, affordable meal.	+
Mulder et al. (2020) [49], USA	Observational, 11 months	Describe national prevalence of workplace policies, practices and interventions to support employee health.	*n =* 338 hospitals	Senior hospital employees responded to the Workplace Health in America survey.	Hospital size and worksite health-promotion factors.	81.7% of hospitals provided health promotion or wellness programmes in the previous year and likelihood of implementation varied based on hospital size (*p* < 0.01) and type (*p* < 0.05). Of those which offered programmes, 53.7% provided healthy diet advice (95% CI, 47.6–59.8%) and 59.9% had programmes to tackle obesity (95% CI, 53.9–65.9%). Of the hospitals which had wellness programmes and contained food outlets, 48.6% displayed nutritional information about calories, sodium or fat (95% CI, 42.3–54.8%), 54.7% used symbols to identify healthy choices (95% CI, 48.4–61.0%) and 19.2% subsidised healthy foods and beverages (95% CI, 14.2–24.1%).	+
Patsch et al. (2016) [24], USA	Quasi-Experimental, 1 year	Assess the impact of subsidisation of healthy food and taxation of unhealthy food on sales and financial outcomes.	*n =* 2800 hospital visitors and employees	Three food items were paired with healthier ‘Better Bites’ alternatives at two hospitals (PH and SFMC). Products were labelled as such and signage was added to canteens. Healthy product cost decreased by 35%, while unhealthy product cost increased by 35%.	Average weekly healthy (Better Bites) and less healthy sales, change in the proportion of healthy and less healthy products sold at each facility and financial outcomes.	At PH, relative traditional burger sale decreased by 47.9% (z = ± 35.85, *p* < 0.001) while the Better Bites option experienced a relative increase of 600% (z = ± 35.85, *p* < 0.001). Better Bites salad sales demonstrated a relative increase of 2.6% (z = ± 1.18, *p* = 0.238). At SFMC, proportion of traditional burger sales experienced a relative decrease of 20.4% (z = ± 14.87, *p* < 0.001) and the Better Bites burger demonstrated a relative increase of 371.2% (z = ± 14.87, *p* < 0.001), but sales remained lower than those of traditional burgers. At this site, Better Bites salad sales showed a relative increase of 71.1% (z = ± 5.32, *p* < 0.001).	∅
Pechey et al. (2019) [38], UK	RCT, 28 weeks	Assess the impact of altering the absolute and relative availability of healthier and less healthy vending machine products.	*n =* 10 hospital food outlets	Vending machines were subjected to five conditions and 20% of items were changed in each condition. Proportion of healthier and less healthy items available was altered each time.	Energy purchased from each vending machine under every condition and number of products vended each week.	Altering the proportion of healthy and unhealthy options did not significantly alter energy purchased from food (decrease less healthy: *p* = 0.407, increase healthier: *p* = 0.103, decrease healthier: *p* = 0.350, increase less healthy: *p* = 0.180). When the number of unhealthy beverages decreased, energy purchased from beverages decreased by 53% (*p* = 0.001). Total sales did not decrease.	+
Public Health England (2018) [39], UK	Quasi-Experimental, 9 months	Assess the impact of nutrition standard implementation and choice architecture on the nutritional quality of vending machine products.	*n =* 17 food outlets	In phase 1, vending machine content was altered to adhere to best practice Government Buying Standards for Food and Catering but healthy items were displayed less prominently. In phase 2, standards were upheld and healthier items were displayed more prominently.	Number of items sold, mean energy content per product and mean sugar content per product.	In drinks machines, total sales increased by 2.5% as fewer sugar-sweetened beverages and more ‘diet’ beverages were sold. Energy per item sold decreased (−36.2%), along with total sugar content (−36.4%). In food machines, overall sales decreased throughout the intervention (−3.2% in phase 1, −11.8% between phase 1 and 2), mainly due to reduced purchase of crisps (−29.1% in phase 1, −23.1% between phase 1 and 2). Confectionary and dried fruit and nuts sales increased in phase 1 (+14.2% and +23.2%) and decreased slightly (but remained above baseline) in phase 2 (−8.2% and −0.8%) and total energy from food decreased in phase 1 (−10.5%) and phase 2 (−9.5%).	∅
Sato et al. (2013) [54], USA	Quasi-Experimental, 12 weeks	Assess the impact of food labelling on the purchase of healthier entrees in cafeterias and on financial outcomes.	*n =* 1 hospital food outlet and *n* = 131 participants surveyed	Following baseline data collection from receipts, labels were added to entrees, displaying information on calorie, fat and sodium content. Customers were surveyed on their usage of these labels.	Change in the nutritional content of purchases pre- and post-intervention and customer preferences on labelling and views regarding the influence of labelling.	Mean percentage of healthier options sold increased by 0.7% while regular menu sales decreased by 0.7% (*p* = 0.837). Overall, total entrée sales decreased by around 8% (*p* < 0.0001) and average price increased by 50 cents. 77% of customers who purchased entrees claimed to have noticed the labels and at least 71% of these respondents expressed positive feelings towards the labels. 50% of those who noticed labels and purchased an entrée claimed that labels influenced their purchase, persuading them to select healthier options.	∅
Sharma et al. (2016) [50], USA	Observational, 2 months	Describe current policies and practices associated with nutrition and physical activity environments in hospitals and compare them between facilities.	*n =* 5 hospitals	The Environmental Assessment Tool was used to assess healthy food availability in six cafeterias and six vending machines. Mean score was calculated at each healthcare facility.	Environmental Assessment Tool factors, such as physical activity and nutrition support.	All hospitals offered nutrition education classes and provided healthy vending machines and cafeteria options. Healthy food availability scores ranged from 62–75% and varied within hospitals. Healthy food availability in vending machines scored 13–36%, while healthy drink availability scored between 0–40%.	∅
Simpson et al. (2018) [40], UK	Quasi-Experimental, 6 months	Assess the feasibility of increasing proportion of healthy options in a hospital shop and the impact on financial outcomes and consumer acceptability.	*n =* 1 hospital food outlet	Portion sizes, promotions, prices, positioning and healthy option availability were adjusted to diminish barriers and implement facilitators to healthy eating. Intervention results were assessed soon after implementation and at a later date.	Relative sales of healthy food products, change in sales within food categories and change in profits.	Adding fruit to the meal deal increased units of fruit sold from 40 to over 900 per week. Total sales increased by 11% but no significant change in relative proportion of healthy food sales. In follow-up, sales increased by 27% but change in relative proportion of healthy food sales remained unchanged. Sales of sweets and chocolate decreased, but sales of other unhealthy products did not. 35% of respondents said the shop sold a good range of healthy options pre-intervention, compared to 60% post-intervention.	∅
Sonnenberg et al. (2013) [25], USA	Quasi-Experimental, 3 months	Assess the impact of product labelling on consumer awareness of healthy purchases.	*n =* 204 (baseline) *n =* 253 (following intervention) hospital employees and visitors	Traffic light labelling and signage were introduced. A dietician was present in the first two weeks of intervention and nutritional information flyers were made available. Consumers were surveyed following purchase of items.	Public perspectives on important factors when purchasing food and beverages, nutritional value of products and the influence of traffic light labelling on purchases.	At baseline, 46% of respondents stated health and nutrition were important factors when making food choices, increasing to 61% following intervention (*p* = 0.004). Taste (*p* = 0.04) and price (*p* = 0.02) also became more important, while convenience became slightly less important (*p* = 0.06). Increased participants reporting usage of nutritional information when making choices (15% to 33%, *p* < 0.001). Those who noticed and were influenced by labels bought more green-labelled items and fewer red-labelled items compared to those who did not (*p* < 0.001).	∅
Stead et al. (2020) [41], UK	Mixed Methods, 18 months (short reassessment 1 year later)	Describe the process of Healthcare Retail Standard implementation and how the standards impact healthy product promotion.	*n =* 17 hospital food outlets	Data on chocolate and fresh fruit was gathered following implementation of the Healthcare Retail Standards (HRS) and interviews were conducted with managers to understand awareness and attitudes regarding the standards.	Number of relevant products on display and number of promotions for relevant products.	12 of 13 shops achieved compliance with the HRS. Mean number of fruit products available did not change, while mean number of chocolate products decreased from an average of 60 to 29. Chocolate promotions in shops decreased from 166 to 38. Managers raised concerns about reduced uptake of meal deals. Allowing introduction of baked crisps into the meal deal slightly increased sales, but not to pre-intervention levels.	∅
Stites et al. (2015) [26], USA	Mixed Methods, 12–16 weeks	Assess the impact of mindfulness and pre-ordering meals on nutritional quality of purchases by hospital employees.	*n =* 26 hospital employees	In the full-intervention, mindful eating educational sessions were combined with encouragement to pre-order canteen meals. Vouchers were also issued. In the partial intervention, vouchers were not issued. Mindful Eating Questionnaires were administered at the beginning and end of the study.	Amount of energy and fat in lunches purchased by employees.	Average calorie content of lunches was 601 kcal and fat content was 4.9 g for the intervention group compared to 745.7 kcal (95% CI = −254.0 to −35.1, *p* = 0.01) and 13.8 g (95% CI = −15.2 to −2.6, *p* = 0.005) for the delayed treatment group. Calorie (95% CI *=* −81.3 to −52.6, *p* < 0.001) and fat content (95% CI = −4.1 to −2.8, *p* < 0.001) also decreased when the financial incentive was removed. Mindful eating behaviours increased from pre- to post-intervention (*p* < 0.001), but weight loss was not statistically significant e (*p* = 0.099). 92% of participants expressed interest in using a pre-ordering system in the future.	+
Sustain (2017) [51], UK	Observational, short duration	Describe current availability of healthy food and drinks in hospitals and determine hospital adherence to nutritional standards.	*n =* 30 hospitals	Surveys were sent to hospitals and assessed fresh food availability, healthy options and access to facilities during breaks.	Information on hospital food standards and types of food available to staff and visitors.	50% of hospitals adhered to all five standards stated in the NHS contract, while 67% reported meeting or working towards health and wellbeing CQUIN targets. 40% of facilities had 24-h access to healthy foods, 25% met the criteria for having a food and drink strategy and 77% offered fresh food to staff. Two hospitals met all of the criteria for healthy food available to staff and visitors and 23 met the goal of having two portions of vegetables per main meal. Six hospitals had 70% or more products in hospital shops with green or orange traffic light labels. Vending machines selling mostly healthy options (*n =* 138) were more prevalent than those selling less healthy options (*n =* 90); 21 hospitals offered meal deals, including healthy options.	∅
Thorndike et al. (2014) [30], USA	Quasi-Experimental, 2 years	Assess the impact of traffic light labelling and choice architecture on hospital cafeteria sales.	*n =* 2285 hospital employees	Traffic light labelling was introduced and, after three months, a choice architecture intervention was also introduced.	Proportion of healthy and unhealthy sales every 3 months.	After one year, proportion of red-labelled items sold decreased from 24% to 21% (*p <* 0.001) and proportion of green-labelled items increased from 41% to 45% (*p* < 0.001). After two years, proportion of red-labelled items sold remained the same, while proportion of green-labelled items increased to 46% (*p* < 0.001). Results were statistically similar among all populations and sales were stable for two years.	∅
Thorndike et al. (2016) [29], USA	RCT, 6 months	Investigate the impact of social norm feedback and financial incentives on nutritional quality of purchases.	*n =* 2672 hospital employees	Participants were randomised to three groups, feedback (monthly comparison to other employee purchases), feedback and incentive (financial reward for healthy purchases) or control	Proportion of healthy items bought at baseline and at end of intervention.	The feedback incentive condition led to a 2.2% in green-labelled purchases (*p* = 0.03) compared to 0.1% for the control. The feedback only condition led to a non-signifcant1.8% increase (*p* = 0.03). There was a significant relationship between health classification at baseline and the impact of interventions on food choices (*p* < 0.001).	+
Thorndike et al. (2019) [27], USA	Observational, 2 years	Investigate the relationship between workplace cafeteria healthy eating programmes and reduced calorie purchases among employees throughout a two year intervention.	*n =* 5695 hospital employees	Sales data was gathered before and after implementation of traffic light labelling.	Calories sold at baseline and end of intervention, plus weight change of employees.	After one year, mean calorie content per transaction decreased by 19 kcal from baseline (95% CI, −23 to −15 kcal, *p* < 0.001). After two years, there had been a mean decrease of 35 kcal, with red-labelled item purchases decreasing by 42 kcal per transaction from baseline (95% CI, −45 to −39 kcal, *p* < 0.001). The dynamic model suggested that frequent users of the hospital cafeteria would lose 1.1 kg in one year and 2 kg in three years as a result of cafeteria interventions, assuming no other changes to eating or exercising behaviour.	+
Thorndike et al. (2021) [28], USA	RCT, 2 years	Assess the impact of an automated behavioural intervention on weight status and nutritional intake of hospital employees.	*n =* 602 hospital employees	Two emails were sent per week, providing feedback on purchasing behaviour and offering personalised advice. 1 letter was also sent per month, comparing participants to peers and offering financial incentives for healthy choices.	Change in weight from baseline to 12 months and 24 months, cafeteria purchases and calories purchased per day.	After 1 year (95% CI, −0.6 to 1.0, *p* = 0.70) and 2 years (95% CI, −0.3 to 1.4, *p* = 0.20), there was no significant difference in weight change between the intervention and control groups. Following the first year, purchases of green-labelled items had increased by 7.3% (95% CI, 5.4 to 9.3) and purchases of red-labelled items had decreased by 3.9% compared to baseline (95% CI, −5.0 to −2.7). Number of calories purchased per day decreased by 49.5 kcal compared to the control group (95% CI, −5.0 to −2.7). Differences remained significant after 2 years. After 1 year, 92% of survey respondents in the intervention group stated that at least one of the intervention methods had supported healthy decision-making.	+
Webb et al. (2011) [55], USA	Quasi-Experimental, 3 months	Gather views of hospital food outlet users and assess the impact of calorie-labelling on purchasing behaviour.	*n =* 6 hospital food outlets and *n =* 554 survey respondents	Cafeterias were subjected to one of three conditions: No labelling, calorie and nutrient labelling on posters and labelling on posters in addition to point-of-purchase menu board labelling.	Attitudes, awareness and usage of calorie information by consumers and the daily number and type of purchases made.	69% of survey respondents using food outlets with menu boards and posters noticed calorie information compared to 58% using outlets with just posters. 32% of respondents noticed calorie information and a third of these participants indicated that it influenced their purchases. Average number of daily purchases remained the same. Proportion of lower-calorie side dishes purchased increased by 4.8% at the menu-board site and decreased by 4.8% at the no labelling site (*p* = 0.0007). Proportion of low-calorie snacks purchased increased by 1.3% at the menu board site and decreased by 8.1% at the no labelling site (*p* < 0.006). Changes to entrée purchases were modest.	∅
Whitt et al. (2018) [42], USA	Quasi-Experimental, 4 months	Compare the impacts of traffic light labelling and cartoon labelling on food choices in a children’s hospital cafeteria.	*n =* 1 hospital food outlet	Products were given a traffic light colour sticker for unhealthy, neutral and healthy, or a Spongebob Squarepants sticker for healthy items.	Proportion of daily healthy, neutral and unhealthy purchases.	Traffic light labelling led to the lowest number of unhealthy food purchases, at 30%, which was significantly lower than the 37% value at baseline (*p* < 0.001). Cartoon labelling increased the number of unhealthy purchases, with a 5% increase from washout (χ^2^ = 5.73 (*p* = 0.057)).	∅
Winston et al. (2013) [52], USA	Observational, 4 months	Assess the impact of socioeconomic status of an area on hospital nutrition composite scores.	*n =* 39 hospitals	The Hospital Nutrition Environment Scan for Cafeterias, Vending Machines and Gift Shops was used to score the hospital food environment.	Barriers and facilitators for a healthy diet.	Average score was less than 25% of possible points and were higher for vending machines (33%) than for cafeterias or gift shops. There was no significant association between socioeconomic status of the area and the nutrition composite score. Less than half of the shelf space in cafeterias was used for healthier items and 85% of cafeterias displayed unhealthy options near the point-of-purchase. Use of a contracted food service was linked to provision of a healthy combination meal and the availability of nutritional information.	∅

+: The report has met targets for quality (such as generalisability, inclusion and exclusion and data collection and analysis) and risk of bias. ∅: Report quality is neither particularly strong nor particularly weak. --: Targets for quality and bias have not been met.
